# Learning From COVID-19: What Would It Take to Be Better Prepared in the Eastern Mediterranean Region?

**DOI:** 10.2196/40491

**Published:** 2024-02-15

**Authors:** Lara Kufoof, Rana Hajjeh, Mohannad Al Nsour, Randa Saad, Victoria Bélorgeot, Abdinasir Abubakar, Yousef Khader, Salman Rawaf

**Affiliations:** 1 Project Management Office Global Health Development, Eastern Mediterranean Public Health Network Amman Jordan; 2 Department of Program Management Regional Office for the Eastern Mediterranean World Health Organization Cairo Egypt; 3 Global Health Development, Eastern Mediterranean Public Health Network Amman Jordan; 4 Department of Research and Policy Global Health Development, Eastern Mediterranean Public Health Network Amman Jordan; 5 Regional Office for the Eastern Mediterranean World Health Organization Cairo Egypt; 6 Department of Public Health Faculty of Medicine Jordan University of Science and Technology Irbid Jordan; 7 Department of Primary Care and Public Health School of Public Health at Imperial College London London United Kingdom

**Keywords:** COVID-19, integration, pandemic preparedness, primary health care, public health

## Abstract

The COVID-19 transmission in the Eastern Mediterranean Region (EMR) was influenced by various factors such as conflict, demographics, travel and social restrictions, migrant workers, weak health systems, and mass gatherings. The countries that responded well to COVID-19 had high-level political commitment, multisectoral coordination, and existing infrastructures that could quickly mobilize. However, some EMR countries faced challenges due to political instability and fragile health systems, which hindered their response strategies. The pandemic highlighted the region’s weak health systems and preparedness, fragmented surveillance systems, and lack of trust in information sharing. COVID-19 exposed the disruption of access and delivery of essential health services as a major health system fragility. In 2020, the World Health Organization (WHO) conducted a global pulse survey, which demonstrated that the EMR experienced the highest disruption in health services compared to other WHO regions. However, thanks to prioritization by the WHO and its member states, significant improvement was observed in 2021 during the second round of the WHO’s National Pulse Survey. The pandemic underscored the importance of political leadership, community engagement, and trust and emphasized that investing in health security benefits everyone. Increasing vaccine coverage, building regional capacities, strengthening health systems, and working toward universal health coverage and health security are all priorities in the EMR. Emergency public health plays a key role in preparing for and responding to pandemics and biological threats. Integrating public health into primary care and investing in public health workforce capacity building is essential to reshaping public health and health emergency preparedness.

## Introduction

The World Health Organization (WHO) was first alerted to cases of pneumonia of unknown origin on December 31, 2019. By January 30, 2020, the WHO had declared the novel coronavirus outbreak a public health emergency of international concern [1]. The WHO’s Director General described this outbreak of COVID-19 as a pandemic on March 11, 2020 [2]. Since then, many countries have announced several restrictive public health measures to contain the virus, such as travel bans, border restrictions, lockdowns, and mandatory quarantines [3]. As a result, global economic growth was severely impacted, with the global gross domestic product dropping by 6.7% in 2020 [4,5]. These measures, along with the strain on resources due to the care of those infected with COVID-19, have disrupted the access to and delivery of essential health services [6]. Even the strongest health systems were heavily impacted and overwhelmed by the pandemic.

In 2020, the WHO conducted a global pulse survey to understand the impact of COVID-19 on health systems. Almost 90% of the 105 engaged countries reported interruptions in different services, ranging from routine and elective service delivery to critical care, especially in low- and middle-income countries [6]. Financial constraints, supply chain disruptions, redirection of services to the care of patients with COVID-19, and workforce unavailability affected access to essential health services [5]. The pandemic revealed that no country was sufficiently prepared against biological threats. Many risks and gaps were identified in the current public health system that hindered countries’ capacities for response. These challenges and gaps called for increased investments and stronger political will to enhance health emergency preparedness [7].

In January 2020, the WHO activated its incident management system at all 3 levels of the organization (global, regional, and country levels), in line with the WHO’s emergency response framework [3]. This system safeguards the coordination of response actions during public health emergencies [3]. The WHO’s Eastern Mediterranean Region (EMR) Incident Management Support Team (IMST) for COVID-19 was activated on January 22, 2020, as a coordination mechanism providing technical, strategic, and operational support to EMR countries. It has been operational for over 2 years as the WHO’s longest-running IMST. On January 29, 2020, the first cases of COVID-19 were reported in the EMR, and by April 10, 2020, all 22 EMR countries and territories had reported COVID-19 cases [8].

The EMR is composed of 22 countries, categorized into 3 groups based on socioeconomic development. Group 1 includes Bahrain, Kuwait, Oman, Qatar, Saudi Arabia, and the United Arab Emirates (UAE), which have the most resources and are all high-income countries. Group 2 includes Egypt, Iran, Iraq, Jordan, Lebanon, Libya, Morocco, Palestine, Syria, and Tunisia, which have the next most level of resources and are upper-middle– or lower-middle–income countries. Group 3 includes Afghanistan, Djibouti, Pakistan, Somalia, Sudan, and Yemen, which have the least resources and are all either lower-middle– or low-income countries [9]. The diversity of country incomes, emergencies, cultures, and health system capacities in the EMR led to varying response capacities, COVID-19 knowledge and risk perceptions, and socioeconomic impacts, which have usually been substantial [10].

This viewpoint highlights the challenges that faced EMR countries and their achievements and lessons learned during the COVID-19 pandemic. It provides an overview of the unequal COVID-19 vaccination coverage in the region and discusses the methods and approaches of how to reshape public health in the region and strengthen health emergency preparedness, providing recommendations for the way forward.

## Challenges in the EMR

Over 50% of the countries in the region are affected by complex emergencies, either directly or indirectly. The EMR holds 9 major humanitarian emergencies, 102 million people needing humanitarian assistance (37% of the global total), and over 32 million refugees and internally displaced persons [8]. Political instability, fragile health systems in some EMR countries, multiple disease outbreaks, and poor accessibility and availability of basic health care services have hampered the effectiveness and efficiency of the strategies adopted to combat COVID-19 [11]. The COVID-19 response in the EMR was influenced by multiple factors affecting viral transmission, such as state fragility and conflict; demographics, as this region has a younger population than most; early applications of travel and social restrictions, which limited spread at the beginning of the pandemic; large numbers of migrant workers; mass gatherings; and pilgrimage. Saudi Arabia made an unprecedented decision to downsize Hajj and suspend Umrah, and other EMR countries implemented public health and social measures. However, as “COVID-19 fatigue” set in, many countries loosened their restrictive measures, which often affected the disease trend [10]. Although some countries used and built on past health emergency experiences and systems, such as Saudi Arabia applying lessons learned from the Middle East respiratory syndrome experience [12,13], other countries were poorly prepared. For example, 6 EMR countries still lack national infection prevention and control guidelines, and 5 (Afghanistan, Iraq, Libya, Palestine, and Tunisia) developed their infection prevention and control guidelines in the past year only, with the support of the WHO [14]. The COVID-19 pandemic has exposed gaps in the health systems at multiple levels globally, even in high-income countries with strong health systems. The Islamic Republic of Iran, for example, which had a strong existing health care system, witnessed the largest rate of infections and deaths [15].

The pandemic also highlighted the weak epidemiological capacity of the region [13]. Data were generated but were not always analyzed, interpreted, and used as evidence for action. Additionally, surveillance systems are fragmented, with many being old and paper based [16]. There is also an issue of trusting the shared information in the region because of the underreporting of some countries or their hesitancy to share data [17,18].

Several countries within the EMR encountered obstacles in addressing the COVID-19 pandemic. For instance, Pakistan struggled due to its fragile health care infrastructure, characterized by shortages of health care professionals, hospital beds, and essential medical equipment necessary for treating patients with COVID-19 [19]. Similarly, Iran encountered difficulties in delivering crucial medical and humanitarian supplies due to economic sanctions [20]. Additionally, Yemen and Syria faced difficulties managing the pandemic within the context of ongoing conflict, displacement, and the challenges of maintaining health care infrastructure and resources [21].

## Achievements and Lessons Learned

Despite the abovementioned challenges, there are many successes and lessons learned. First, the EMR countries that succeeded in facing COVID-19 had high-level political commitment and high-level multisectoral coordination. Often, the heads of state or prime ministers led the multisectoral committees or crisis committees, and this is an example of leadership. Also, regional laboratories were quick to mobilize and build on previous infrastructure and systems, such as those dedicated to influenza, polio, and other communicable diseases [10,22]. Polio response teams were used in the field and played major roles in COVID-19 vaccination and community mobilization [22]. Before the COVID-19 pandemic, the region had expanded its influenza network due to the influenza pandemic potential. The WHO supported the strengthening of influenza surveillance and testing for respiratory infections. As a result, most countries had the capacity to test for respiratory viruses in a timely manner, allowing the successful use of reverse transcription polymerase chain reaction (RT-PCR) tests for SARS-CoV-2 testing in influenza labs and national influenza centers [23]. RT-PCR capacity for SARS-CoV-2 was expanded quickly to subnational levels across all 22 EMR countries. The WHO ensured the quality of SARS-CoV-2 testing by encouraging all countries to participate in the external quality assessment program for national and subnational laboratories. The WHO’s logistics hub in Dubai, UAE, which is the WHO’s largest repository of medical equipment and supplies globally, was also an asset to the region as it moved thousands of tons of supplies to the world and the region, including millions of RT-PCR tests [24].

Due to a lack of high-quality data, epidemiologists had to consider a variety of indicators [25], including testing rates and measuring the burden on health systems-related indicators, such as hospital occupancy, intensive care unit occupancy, bed occupancy rates, and case fatality ratios. These indicators allowed the WHO to estimate the extent of the pandemic and the COVID-19 response. A very high case fatality ratio could mean that the country is not reporting all cases, has a weak testing capacity or strategy, is poorly managing cases, or may have different case fatality definitions. Therefore, it is important to not only report data but to also interpret it for evidence-based policy making [25].

Limited testing capacity, supplies, and infrastructure were other reasons countries did not provide accurate data [26]. For example, Somalia and Djibouti did not have RT-PCR capacity at the beginning of the pandemic; however, they managed to procure the needed equipment [27,28]. Enhanced data reporting was significantly increased due to improved laboratory capacity in the region, both for testing and sequencing. The pandemic was an opportunity to build capacity in genome sequencing, enabling the monitoring of the circulation of SARS-CoV-2 variants. Almost 15 countries in the region have genome sequencing capacity and are able to support other countries without such capacity [16,29].

The pandemic resulted in several innovations, including apps, telemedicine, hotlines, and e-clinics. More countries in the region used innovative solutions to improve data collection, analysis, and dissemination to build strong surveillance capacities and bridge information gaps. For example, using phone companies’ mobility data to measure whether social interventions are successfully implemented allowed for a better understanding of people’s mobility patterns [30]. Another innovation included oxygen production and supply, as some countries, such as Somalia, had no oxygen plants and no capacity to produce oxygen [31]. Since Somalia did not have electric power, especially at the subnational level, they built small solar plants and used solar power to generate oxygen [31]. Similarly, there was a lot of stigma around mental health in the region [32]. However, during the pandemic, 17 countries (Afghanistan, Bahrain, Egypt, Iran, Iraq, Jordan, Kuwait, Lebanon, Libya, Morocco, Pakistan, Palestine, Qatar, Saudi Arabia, Tunisia, UAE, and Yemen) incorporated mental health and psychosocial support within their emergency response plans by establishing hotlines, e-clinic consultations, and platforms to serve the remote areas [33-35].

A total of 17 EMR countries (Bahrain, Egypt, Iran, Iraq, Jordan, Kuwait, Lebanon, Oman, Pakistan, Palestine, Qatar, Saudi Arabia, Sudan, Syria Arab Republic, Tunisia, UAE, and Yemen) banned waterpipe smoking outdoors; however, this was temporary [36]. In terms of the next steps and moving forward, the region needs to take advantage of these innovations and opportunities to enhance preparedness because they are feasible if there is the right commitment and policies.

There was a need for epidemiological modeling to predict the potential impact of public health and social measures, so the WHO established a modeling support team and used data from countries that requested this assistance to help model the progression of the pandemic [37]. COVID-19 also increased interest in epidemiological studies on the ground, including seroprevalence studies to understand population immunity levels or risk factors. The WHO provides standard protocols for seroprevalence studies [38]. The WHO also worked with countries on vaccine effectiveness studies, and many countries took part in clinical trials on vaccines and therapeutics. Through this, countries built their capacities, which is helpful for preparedness [16].

The positive side of the COVID-19 pandemic was that it revealed many of the gaps and weaknesses in the public health system and how to bridge these gaps [7]. The gaps include the nonflexibility of the health systems, workforce shortages, health service fragmentation (primary care, secondary care, and public health), and designs of health facilities. Additionally, misinformation was a significant challenge during the pandemic, and the outdated “disease model” adopted by most health systems does not meet the current needs of the population [39]. Indeed, access and use of essential health services such as maternal and neonatal care and routine immunization were highly affected [40]. The WHO conducted 3 surveys [41,42] to measure the global disruption of essential health services. The first, conducted between May 2020 and July 2020, showed that the EMR was the WHO region with the highest disruption in health services due to health center closures, stigma, and fear of transmission [41]. The WHO worked closely with countries and published guidelines to address the impact of the pandemic on essential health services, including how primary health care workers should be trained to screen for COVID-19 [40]. The same survey conducted a year later, in April 2021, demonstrated a significant improvement in the continuity of essential health services, although they were still disrupted [42].

## COVID-19 Vaccination

With regard to vaccination, increasing vaccine coverage is a WHO priority for some EMR countries. In less than a year, effective vaccines were developed, tested, produced, and administered, which is an unprecedented success in the history of infectious diseases [43]. The goal of the WHO for the EMR is to reach 40% coverage by the end of 2021 and 70% by mid-2022. A total of 9 countries (Bahrain, Iran, Jordan, Kuwait, Morocco, Oman, Qatar, Saudi Arabia, Tunisia, and the UAE) have so far achieved the end-year goal; however, 6 conflict-affected countries (Afghanistan, Djibouti, Somalia, Sudan, Syria, and Yemen) are at less than 10% coverage as of December 2021 [44,45]. As of October 21, 2023, a total of 13,533,465,652 vaccine doses had been administered worldwide. Globally, the persons vaccinated with a complete primary series per 100 population and the persons vaccinated with at least 1 booster or additional dose per 100 population were 66.18 and 31.9, respectively. In the EMR, the respective figures were 51.61 and 19.0, respectively. Within the EMR, the figures were lower, with 51.61 individuals per 100 population having completed a primary series and 19.0 individuals per 100 population having received at least one booster or an additional dose [46].

## Recommendations from the World Health Assembly That Benefit the EMR

Many committees presented their recommendations during the last World Health Assembly, including the Independent Panel for Pandemic Preparedness (established by the WHO and Global Preparedness Monitoring Board) and the Independent Oversight Committee. The committees came up with over 200 recommendations [47], but some of the key recommendations and lessons learned that will benefit the EMR include the following:

1. The importance of political leadership is what made a big difference in the countries that did better than others.
2. Community engagement and community trust are important to prevent the pandemic’s spread and major economic collapse.
3. “No one is safe until everyone is safe” is not just a slogan, because if any country does not implement prevention measures, the virus can travel to other areas of the world.
4. The return on investment in health security is immense. How long does it take to receive a return on your investment? How much do we need to invest in pandemics? There were estimates done, and an early estimate of the cost of the pandemic was about US $11 trillion up to the middle of 2021; another US $10 trillion has been added since then. However, if you invest in preparedness, it will cost US $5 per person per year, so for a global population of 8 billion, it will only cost US $40 billion. To prompt action from policy makers, it is essential to present them with these monetary figures.

## Reshaping Public Health

During the COVID-19 pandemic, different reasons have called on the EMR countries to reshape their public health. Many countries in the EMR are experiencing new dynamic population growth due to birth, migration, and aging populations. Additionally, the region is facing what is termed a “brain drain.” For example, Egypt has a shortage of doctors because 65% of Egyptian doctors leave the country to seek opportunities abroad. Additionally, the world is witnessing continuous technological advances in the biological, physical, and digital spheres [48].

The burden of the disease in the region should also be considered. The Institute of Health Metrics studied the number of deaths per 100,000 in 2019, per disease per country, and showed that besides the known causes of death such as communicable disease, noncommunicable disease, etc, the region has witnessed other causes of mortality due to violence, mainly in Iraq, Syria, and Yemen. Accordingly, in the region, the leading cause of death from 2000-2019 was related to collective violence and interventions [49].

Furthermore, there is no specific data on public health personnel in the region. Data are mainly available on medical health workers, and Saudi Arabia and Sudan are the only 2 countries with registered public health professionals in the Arab world. On the other hand, only a small proportion of the public health workforce (public health consultants) are fully trained with 4-5 years of competency-based training, and around one-quarter (public health practitioners) are qualified in public health but without structured training. Therefore, the public workforce in the region needs to be tackled in terms of skills, experience, and foundational knowledge [50].

Investments in primary health care to mitigate the risks of future pandemics and to maintain accessibility and delivery of essential health services during emergencies [40]; investment in the health workforce, including training, mobilization, and redistribution to sustain high-quality essential health services delivery; and data management and surveillance are key elements of a successful response [16]. These actions and initiatives can also expand and reinforce health system capacities, providing an opportunity within the COVID-19 response for countries to reshape their health workforce and services and improve health security for future health emergencies [7,40,51].

Integrating public health into primary health care is an essential approach to reshaping public health and achieving preparedness. Primary care is the first point of contact for the community with the health system; it must be available 24 hours a day and should provide services in a continuous, personalized, and holistic way. Unfortunately, in many settings, primary health care focuses on treating the illness rather than preventing it. Therefore, integrating public health functions into the primary health care system is highly significant to ensure disease prevention, health promotion and protection, and a proper response to threats [39].

Accordingly, 6 models were identified by the WHO technical series on primary care called “Closing the gaps between public health and primary care through integration” to attain the integration of public health into primary health care and provide a tool to help countries be prepared during threats and emergencies. This, in turn, focuses the services on the population’s needs, achieving a person-centered approach. These models can be applied either individually or in combination, depending on the flexibility of the health systems, and they are titled as follows [39]:

1. Public health services are integrated into primary care
2. Public health professionals and primary care providers are working together
3. Comprehensive and proactive benefit packages that include public health
4. Primary care services within public health settings
5. Building public health incentives in primary care
6. Multidisciplinary training of primary care staff in public health

With a focus on the first model, integrating public health professionals into primary care, where they are involved in many public health functions, can be easily achieved progressively. It is essential to understand that the primary health team is complex and should not only include family medicine and freshly graduated doctors; rather, it should include the public health workforce, such as Field Epidemiology Training Program professionals. Additionally, building the competencies of the public health workforce is crucial because, even though the region has 2 public health academies—the International Academy of Public Health and Weqaya Public Health Academy—most of the workforce is untrained [39,50].

Many aspects need to be considered when reshaping public health: (1) financial allocation and establishing an independent national body for public health; (2) investing in public health laboratories, whole genome sequencing, public health analysis such as artificial intelligence, real-world data, real-world evidence, research, and people; and (3) teaching for precision public health where people are the center of health.

A hard lesson learned from this pandemic is that countries should not be dependent on other countries to provide them with essential medicines and vaccines. It is of great importance to strengthen health systems and work toward universal health coverage and health security, as Dr Tedros Adhanom Ghebreyesus, the Director General of the WHO, says that “Health security and global health coverage are two sides of the same coin” [52]. Capacity building is the way forward, including capacity building in integrated disease surveillance and in ensuring the continuity of health services. Additionally, engaging all relevant stakeholders, accelerating vaccine rollout, prioritizing COVID-19 response, and investing in emergency preparedness and the health systems are essential.

## Conclusion

The political instability and fragile health systems in some of the EMR countries have hampered the effectiveness and efficiency of the strategies adopted to combat the COVID-19 pandemic. The EMR IMST for COVID-19 pillars was critical to the WHO’s role in coordinating the response during the pandemic. Although multiple challenges affect the transmission of the virus in this diverse region, there were many successes, and it is of great importance to build on these successes and focus on building the human and regional capacities as a way forward. Furthermore, focusing on public health is a key factor in responding to pandemics and biological threats, with COVID-19 being a clear example. The current health system faces many gaps and challenges, which can be overcome by adopting different approaches—specifically, integrating public health into primary care as an essential approach to reshape public health in the region and be prepared against threats and emergencies.

## Recommendations and Areas of Improvement

Several areas of improvement need to be taken into consideration at both the national and regional levels to improve the response to future threats and pandemics. Countries should develop and update a multisectoral emergency preparedness plan and enhance government and political leadership capacity toward biological threats. They must strengthen their health systems and work toward universal health coverage and security. This can be achieved by integrating public health into primary care as an essential approach to reshaping public health through adopting 1 or more of the 6 models of integration identified by the WHO. Moreover, there is a need to invest in building human capacities, including epidemiologists; emergency responders; community health workers; health economists; communication specialists; and most crucially, health leaders. Countries should also work toward community engagement and community trust by assessing people’s needs and engaging them in the decision-making process because public health is about people, for people, and by people. Finally, analyzing and interpreting collected data and using it by policy makers is essential for action and decision-making.

On the other hand, different actions need to be taken at the regional level to effectively control the spread of the pandemic. As the COVID-19 pandemic demonstrated, certain countries have greater capacities than others in the region and must facilitate cooperation, solidarity, and support. High-income countries, for example, should ensure vaccine sharing, equity, and distribution with low-income countries. Moreover, countries in the region can implement twin programs where human resources can be shared across countries.

## Abbreviations

EMR: Eastern Mediterranean Region
IMST: Incident Management Support Team
RT-PCR: reverse transcription polymerase chain reaction
UAE: United Arab Emirates
WHO: World Health Organization
